# Supplementary material for: Error-based or target-based? A unified framework for learning in recurrent spiking networks
Source: PLoS Comput Biol. 2022 Jun 21;18(6):e1010221. doi: 10.1371/journal.pcbi.1010221 (PMC9249234; doi:10.1371/journal.pcbi.1010221)
Supplement: S1 Text — (PDF) [file pcbi.1010221.s001.pdf]

Supporting Information

Error-based or target-based? A unified framework for learning in recurrent spiking networks

A Model and learning rule

A.1 The spiking model

In our formalism, neurons are modeled as real-valued variable $v_j^t \in \mathbb{R}$, where the $j \in \{1, \dots, N\}$ label identifies the neuron and $t \in \{1, \dots, T\}$ is a discrete time variable. Each neuron exposes an observable state $s_j^t \in \{0, 1\}$, which represents the occurrence of a spike from neuron j at time t. We then define the following dynamics for our model:

$$\hat{s}_i^t = \exp\left(-\frac{\Delta t}{\tau_s}\right) \hat{s}_i^{t-1} + \left(1 - \exp\left(-\frac{\Delta t}{\tau_s}\right)\right) s_i^t \tag{1}$$

$$v_i^t = \exp\left(-\frac{\Delta t}{\tau_m}\right) v_i^{t-1} + \left(1 - \exp\left(-\frac{\Delta t}{\tau_m}\right)\right) \left(\sum_j w_{ij} \hat{s}_j^{t-1} + I_i^t + v_{\text{rest}}\right) - w_{\text{res}} s_i^{t-1} \tag{2}$$

$$s_i^{t+1} = \Theta\left[v_i^t - v^{\text{th}}\right] \tag{3}$$

Δt is the discrete time-integration step, while τ_s and τ_m are respectively the spike-filtering time constant and the temporal membrane constant. Each neuron is a leaky integrator with a recurrent filtered input obtained via a synaptic matrix $w \in \mathbb{R}^{N \times N}$ and an external signal I_i^t. $w_{\text{res}} = -20$ accounts for the reset of the membrane potential after the emission of a spike. $v^{\text{th}} = 0$ and $v_{\text{rest}} = -4$ are the the threshold and the rest membrane potential.

A.2 Error-based learning rule, complete derivation

We derive here the expression for the synaptic update (eq. (6) in the main text) that is obtained in the error-based framework for the minimization of an output error E. We assume a regression problem where the error is computed as:

$$\text{E} = \sum_{t,k} \left(y_k^{\star t} - y_k^t\right)^2, \tag{4}$$

moreover we assume the system output $y_k^t \in \mathbb{R}$ to be a linear readout (via readout real matrix B_{ik}) of the low-pass filtered network activity $\bar{s}_i^t$:

$$y_k^t = \sum_i \mathsf{B}_{ik} \bar{s}_i^t \tag{5}$$

where $\bar{s}_i^t$ can be evaluated iteratively as:

$$\bar{s}_i^t = \beta_{\text{RO}}\, \bar{s}_i^{t-1} + (1 - \beta_{\text{RO}})\, s_i^t. \tag{6}$$

with $\beta_{\mathrm{RO}} = \exp\left(-\Delta t/\tau_{\mathrm{RO}}\right)$. The resulting formulation for the synaptic update Δw_{ij} is obtained by imposing it to be proportional to the negative error gradient, with the proportionality factor η representing the learning rate of the system. Following a classical factorization of the error gradient computation in recurrent network, one start by noticing how the time-unrolled network has a feed-forward structure with layers indexed by the time variable t and shared weights $w_{ij}^t = w_{ij}\ \forall t$. The gradient for a specific time-layer can be expressed as (we use the superscript $w_{ij}^{(t)}$ to denote the formal dependence of the synaptic matrix on the layer of time-unrolled, however being the network recurrent, we will omit this trivial index in subsequent expressions):

$$\frac{d\mathrm{E}}{dw_{ij}^{(t)}} = \frac{d\mathrm{E}}{dv_i^t}\frac{\partial v_i^t}{\partial w_{ij}^{(t)}}. \tag{7}$$

The total gradient is obtained by summing the contributions from all the time-layers, yielding the following expression for the error-gradient synaptic update:

$$\Delta w_{ij} = -\eta\frac{d\mathrm{E}}{dw_{ij}} = -\sum_t \frac{d\mathrm{E}}{dv_i^t}\frac{\partial v_i^t}{\partial w_{ij}}. \tag{8}$$

Following [2], we rewrite the error total derivative by collecting all the terms that can be computed locally. The core issue is the fact that the error $\mathrm{E} = \mathrm{E}\left(s^1, s^2, \ldots, s^T\right)$ (with $s^t = \left\{s_i^t\right\}, \forall i$), is a function of the complete network activity, so the influence of a spike s_i^t on the subsequent network development should be backtracked in the computation of the total derivative. We aim for a recursive rewriting by noting that:

$$\frac{d\mathrm{E}}{dv_i^t} = \frac{d\mathrm{E}}{ds_i^{t+1}}\frac{\partial s_i^{t+1}}{\partial v_i^t} + \frac{d\mathrm{E}}{dv_i^{t+1}}\frac{\partial v_i^{t+1}}{\partial v_i^t}. \tag{9}$$

Indeed the second term is suited for an analogous manipulation. The recursive chain terminates for v_i^{T+1}, having the error no dependence for such variable. Applying this strategy yields the following expression for the error gradient:

$$\frac{d\mathrm{E}}{dw_{ij}} = \sum_t \left[\frac{d\mathrm{E}}{ds_i^{t+1}}\frac{\partial s_i^{t+1}}{\partial v_i^t} + \left(\frac{d\mathrm{E}}{ds_i^{t+2}}\frac{\partial s_i^{t+2}}{\partial v_i^{t+1}} + [\ldots]\frac{\partial v_i^{t+2}}{\partial v_i^{t+1}}\right)\frac{\partial v_i^{t+1}}{\partial v_i^t}\right]\frac{\partial v_i^t}{\partial w_{ij}}. \tag{10}$$

Collecting all the terms of the form $\partial v_i^{t+1}/\partial v_i^t$ yields the following compact expression for the gradient:

$$\frac{d\mathrm{E}}{dw_{ij}} = \sum_\tau \sum_{t>\tau} \frac{d\mathrm{E}}{ds_i^{t+1}}\frac{\partial s_i^{t+1}}{\partial v_i^t}\left[\frac{\partial v_i^t}{\partial v_i^{t-1}}\cdots\frac{\partial v_i^{\tau+1}}{\partial v_i^\tau}\right]\frac{\partial v_i^\tau}{\partial w_{ij}}. \tag{11}$$

The current form still bares the issue of require computation of future event (terms indexed by $t > \tau$). However this problem is only apparent (see [2]), as it can be solved by exchanging the summation indices and rewriting the former expression in a form that, at each time t, only involves past events (thus being physically plausible):

$$\frac{d\mathrm{E}}{dw_{ij}} = \sum_t \frac{d\mathrm{E}}{ds_i^{t+1}}\frac{\partial s_i^{t+1}}{\partial v_i^t}\sum_{\tau<t}\left(\frac{\partial v_i^t}{\partial v_i^{t-1}}\cdots\frac{\partial v_i^{\tau+1}}{\partial v_i^\tau}\right)\frac{\partial v_i^\tau}{\partial w_{ij}}. \tag{12}$$

If we recognize in the last term $\partial v_i^t/\partial w_{ij} = (1-\beta_m)\,\hat{s}_j^t$ and note how $\partial v_i^t/\partial v_i^{t-1} = \beta_m$, where $\beta_m = \exp\left(-\Delta t/\tau_m\right)$, the second summation in the gradient is recognized as a low-pass filter of $\hat{s}_j^t$, which yields the spike response function, namely:

$$e_j^t = \sum_{\tau<t} \beta_m^{t-\tau} \left(1-\beta_m\right) \hat{s}^t. \tag{13}$$

We stress that this formulation is equivalent to eq. (7) of the main text. Inspecting the term $\partial s_i^{t+1} / \partial v_i^t$ we note how, according to eq. (3) of the main text, the spike s_i^t depends in a non-differentiable way on the neuron voltage v_i^t (via $\Theta\left[v_i^t - v^{\text{th}}\right]$). This is a fundamental characteristic of spike-based system, which is usually dealt with the introduction of a custom, non-linear pseudo-derivative p_i^t (see eq. (8) of main text). With the previous substitution in place the gradient reads:

$$\frac{d\mathrm{E}}{dw_{ij}} = \sum_t \frac{d\mathrm{E}}{ds_i^{t+1}} p_i^t e_j^t. \tag{14}$$

Up until this point all the manipulation have yielded an exact expression. However, the computation of the total derivative of the error with respect to the neuron spike still needs to be accounted for. Again we face the problem of the cascading influence of the term s_i^t on the entire future network activity. In [2] the following approximation is introduced:

$$\frac{d\mathrm{E}}{ds_i^t} \simeq \frac{\partial \mathrm{E}}{\partial s_i^t}, \tag{15}$$

where the symbol ∂ indicates that only direct contributions of s_i^t on E should be accounted for in the derivation, thus removing the influences of spike s_i^t on subsequent network activity (i.e. the elicit of spike $s_j^{t_+}$ for $t_+ > t$). This approximation is mandatory for a biologically plausible learning rule, which must satisfy space-time locality. If we substitute the approximation (15) into eq. (14) (and use the explicit expression for the error E in (4) and (5)) we get:

$$\frac{d\mathrm{E}}{dw_{ij}} \simeq \sum_t \frac{\partial \mathrm{E}}{\partial s_i^t} p_i^t \hat{e}_j^t = 2\left(\beta_\star - 1\right) \sum_{t,k} \left[\sum_{\tau>t} \mathsf{B}_{ik} \left(y_k^{\star\tau} - y_k^\tau\right) \beta_\star^{\tau-t}\right] p_i^t e_j^t, \tag{16}$$

where $\beta_\star = \exp\left(-\Delta t / \tau_\star\right)$. Again, the apparent issue of the sum on future events can be solved by an exchange in the summation indices, which yields:

$$\frac{d\mathrm{E}}{dw_{ij}} \simeq 2\left(\beta_\star - 1\right) \sum_{t,k} \mathsf{B}_{ik} \left(y_k^{\star t} - y_k^t\right) \sum_{\tau<t} \beta_\star^{t-\tau} p_i^\tau e_j^\tau. \tag{17}$$

The previous expression for the error gradient can then be used as a synaptic update rule to improve network performances. In our experiments we have introduced an additional approximation to (17) by neglecting the following temporal filter:

$$\sum_{\tau<t} \beta_\star^{t-\tau} p_i^\tau e_j^\tau \simeq \beta_\star p_i^{t-1} e_j^{t-1}. \tag{18}$$

These additional approximations justify the use of the $\simeq$ symbol in eq. (6) of the main text and yield our final expression for the synaptic update rule (where all constant factors are included in the definition of the learning rate η):

$$\Delta w_{ij} \simeq \eta \sum_t \left[\sum_k \mathsf{B}_{ik} \left(y_k^{t+1} - y_k^{t+1}\right)\right] p_i^t e_j^t. \tag{19}$$

Indeed, we observe that this approximation does not significantly affect the learning. It is straightforward to derive the learning rule for the readout weights:

$$\Delta B_{kj} = -\eta_{\mathrm{RO}} \frac{d\mathrm{E}}{dB_{kj}} = \eta_{\mathrm{RO}} \sum_t \left(y_k^{\star t} - y_k^t \right) \bar{s}_j^t. \tag{20}$$

B Dimensionality of solution space

This section provides more details about the section *Dimensionality of solution space* and Fig.2 of the main text.

B.1 Store and recall of a 3D trajectory and training protocol

To investigate the properties of the solution space, we decided to store and recall a 3D continuous trajectory. Given a target input x_h^t ($h = 1, ..I$, $t = 1, ..T$), the network should reproduce the target output $y_k^{\star\ t}$ ($k = 1, ..O$, $t = 1, ..T$). $y_k^{\star\ t}$ is a temporal pattern composed of 3 independent continuous signals. Each target signal is specified as the superposition of the four frequencies $f \in \{1, 2, 3, 5\}$ Hz with uniformly extracted random amplitude $A \in [0.5, 2.0]$ and phase $\phi \in [0, 2\pi]$. In this case the input, referred to as clock signal, is defined as follows. For $t \in (0, 0.2\,T]$, $x_1^t = 1$ while the other units are zero. For $t \in (0.2\,T, 0.4\,T]$, $x_2^t = 1$ while the other units are zero and so on. The general rule can be written as $x_k^t = 1$ if $t \in (0.2(k-1)\,T, 0.2k\,T]$, $x_k^t = 0$ otherwise. In order to train our recurrent spiking network, the first step is to compute the target pattern of spikes $s_i^{\star t}$ is computed as:

$$\hat{s}_i^{\star t} = \exp\left(-\frac{\Delta t}{\tau_s}\right) \hat{s}_i^{\star t-1} + \left(1 - \exp\left(-\frac{\Delta t}{\tau_s}\right)\right) s_i^{\star t} \tag{21}$$

$$v_i^t = \exp\left(-\frac{\Delta t}{\tau_m}\right) v_i^{t-1} + \left(1 - \exp\left(-\frac{\Delta t}{\tau_m}\right)\right) \left(\sum_j w_{ij} \hat{s}_j^{\star t-1} + I_i^t + v_{\mathrm{rest}} \right) - w_{\mathrm{res}} s_i^{\star t-1} \tag{22}$$

$$s_i^{\star t+1} = \Theta\left[v_i^t - v^{\mathrm{th}} \right] \tag{23}$$

where $I_i^t = I_i^{\mathrm{teach},t} + I_i^{\mathrm{in},t}$. $I_i^{\mathrm{teach},t} = \sum_h w_{ih}^{\mathrm{teach}} y_h^{\star t+1}$ and $I_i^{\mathrm{in},t} = \sum_h w_{ih}^{\mathrm{in}} x_h^{\star t}$. This term is only used to compute the target, and is not present during the test. $w_{ih}^{\mathrm{in},t}$ and $w_{ik}^{\mathrm{teach},t}$ are random matrix whose elements are randomly drown from a Gaussian distribution with zero means and respective variances σ_{in} and σ_{teach}. All the parameters are reported in Table A.

The recurrent weights are learned with the following rule (eq.(14) of the main text)

$$\Delta w_{ij} = \eta \sum_t \left[\sum_k \mathsf{D}_{ik} \left(\bar{s}_k^{\star t+1} - \bar{s}_k^{t+1} \right) \right] p_i^t e_j^t, \tag{24}$$

while the learning rule for the readout weights is defined in eq.(20).

	Network Parameters	
Name	Description	Value
N	Number of units	100
T	Expert demonstration duration	100
I	Number of input units	5
O	Number of output units	3
dt	Time step	1ms
τ_m	Membrane time scale	8 dt
τ_s	Synaptic integration time scale	2 dt
τ_{RO}	Readout time scale	20 dt
δv	Pseudo-derivative width	0.2
v_{rest}	Rest membrane potential	-4
	Training Parameters	
σ_J	Variance of initial weights	0
σ_{in}	Variance of input matrix	30
σ_{teach}	Variance of teach signal	1.0
η	Recurrent learning rate	0.1
η_{RO}	Readout learning rate	0.015
epochs	Training iterations	1000

Table A: Parameters for the Store & Recall

B.1.1 Tolerance to spike timing

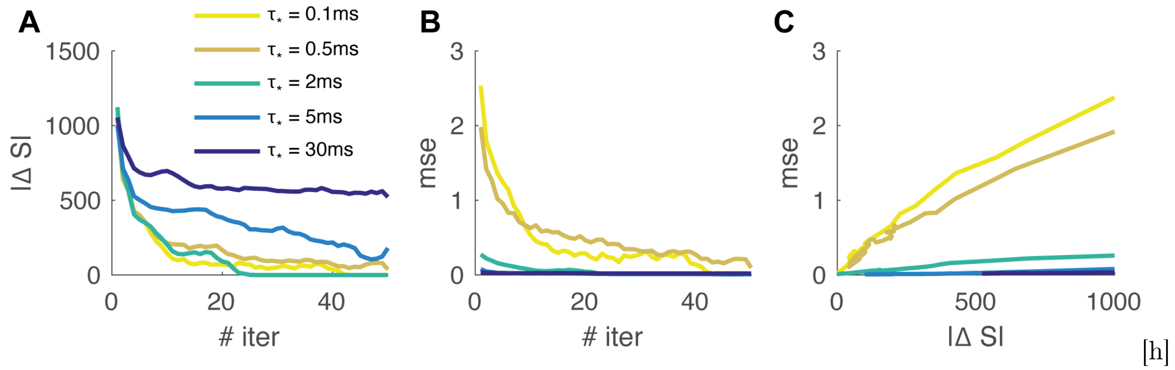

Figure A: **Target-based learning for different time-scales.** (**A**). Color-coded the error on the spike sequence $\Delta S = \sum_{it} |s_i^{\star\ t} - s_i^t|$ as a function of the number of iterations for different $\tau_\star$. (**B**). Color-coded (as in A) the mse $= \sum_{kt}(y_k^{\star\ t} - y_k^t)^2$ as a function of the number of iterations for different $\tau_\star$. (**C**). Color-coded (as in A) the mse as a function of ΔS for different values of $\tau_\star$.

As we discussed above, the $\tau_\star$ can be interpreted as the tolerance to precise spike timing. To further investigate the role of this parameter, we considered the same store and recall task of a 3D trajectory described in the previous section ($N = 100$, $T = 100$). We set the maximum rank ($\mathcal{D} = N$) for this experiment. In

Fig.3A we report the spike error $\Delta S = \sum_{it} |s_i^{\star\, t} - s_i^t|$ as a function of the iteration number for different values of the parameter $\tau_\star$. Only for the lower values of $\tau_\star$ the algorithm converges exactly to the spike pattern $s_i^{\star\, t}$. In Fig.3B we report the mse $= \sum_{kt}(y_k^{\star\, t} - y_k^t)^2$ as a function of the iteration numbers and the parameter $\tau_\star$. In Fig.3C we show the mse as a function of ΔS for different values of $\tau_\star$. Lower $\tau_\star$ values are characterized by a higher slope, meaning that a change in the spike pattern expressed by the network strongly affects the error on the output y_k^t. This suggests a low tolerance to precise spike timing in the generated output when the parameter $\tau_\star$ is low. The consequence of this effect in a behavioral task is investigated in the section "2D Bipedal Walker".

B.2 PC representation and dimensionality estimation

The learning dynamics can be easily pictured as a trajectory where a single point is a complete history of the network activity $s_n = \{s_i^t : i = 1, ...N; t = 1, ...T\}$. For simplicity, and for visualization purposes we defined each point of the trajectory as defined by the vector $s_n := \sum_t |s_i^{\star\, t} - s_i^t|$. Every 50 learning steps a vector s_n is collected. 10 different realizations of the experiment are performed (for different initialization of the initial recurrent weights, which are randomly extracted from a Gaussian with zero mean and variance 2.0). This procedure provides 10 different trajectories s_n. The first 2 PCs of these trajectories are reported in Fig.2A-B of the main text.

B.3 Dimensionality estimation

In order to estimate the dimensionality of the solution space we consider the difference between the activity generated by the network at the end of the learning procedure and the target sequence $\delta s_i^t = s_i^{\star\, t} - s_i^t$.

When the sequence is perfectly cloned, $\delta s_i^t = 0$ by definition. Otherwise these deviations are different from zero. In order to estimate the dimensionality of the sub-space of the solution containing δs_i^t we first perform the PC analysis. PCA is applied on a collection of $T = 100$ vectors δs_i^t each of them defined by $N = 100$ coordinates. As a result we obtain N principal component variances λ_k. If only one variance is significantly different from zero the dimensionality is approximately one, and so on. The dimensionality can be estimated as $d = \frac{(\sum_k \lambda_k)^2}{\sum_k \lambda_k^2}$.

B.4 Random D matrix

We repeated the experiment of the main text (reported in Fig.2C) for the case in which the matrix D_{ik} is random. Its elements are randomly extracted from a Gaussian with mean zero and variance $\frac{1}{\sqrt{\mathcal{D}}}$. The result, reported in Fig.SB is analogous with what observed in the paper.

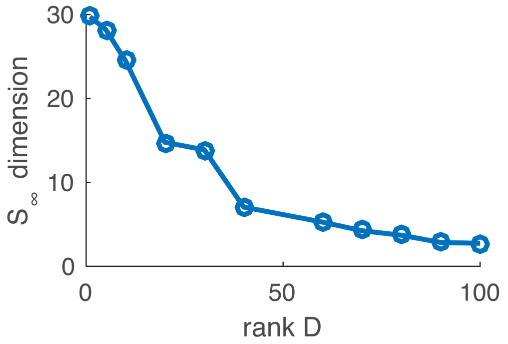

Figure B: **Dimensionality of the solution space: D random.** Dimensionality of the solution space $\mathcal{S}_\infty$ as a function of the rank R of the error propagation matrix.

C Button-and-food

C.1 Training details

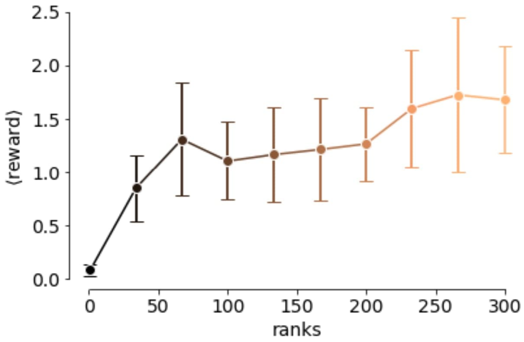

Figure C: **Button & Food: R random.** (**A**) Average reward as a function of the test angle θ in the Button & Food task. Purple arrows mark the training angles, while different colors code for different ranks of the feedback matrix D_{ik}. (**B**) Average reward across all test angles as a function of the rank of the feedback matrix D_{ik}. Error bars are standard deviations over approximately 100 repetitions of the experiment. We used $N = 300$ and $T = 80$.

The Button & Food task requires an agent, which starts at the center of the environment domain, to reach for a button in order to unlock its final target (the food) which sits on a separate location. For this task, we used the same learning procedure as described in Appendix B.1. In particular, in the main text in Fig.4 of the main text, we reported results obtained when training using the semi-clamped version of the learning rule. In the clamped version one substitutes the target spiking activity $s_i^{\star t}$ to the network activity s_i^t during the evaluation of the spike response function e_j^t, indeed, upon convergence, learning halts precisely when $s_i^t = s_i^{\star t}$. However, when $\mathcal{D} < N$ and D_{ik} is diagonal, the target cannot effectively be enforced (or learned) for the $N - \mathcal{D}$ κ-neurons for which $\mathsf{D}_{\kappa\kappa} = 0$, so $s_i^{\star t}$ is only replaced to s_i^t for $i \neq \kappa$, yielding the semi-clamped formulation.

In our experiments, we set the initial agent position at coordinates $(x, y) = (0, 0)$, with the button positioned at $(0, -0.2)$. The target sits at a constant radius ($R_\star = 0.7$) from the origin, while the angle $\theta_\star$ is varied in the range $[30°, 150°]$. The agent is trained on the angles $\theta \in \{50, 70, 90, 110, 130\}$ and tested on the complete range. We fix the network size and task temporal duration to $N = 300$ and $T = 80$ respectively. The expert trajectories are straight lines connecting agent initial location to button to food, travelled with constant speed so to match the travel time of $T^1_{a\to b} = 30$ and $T^2_{b\to f} = 50$. Additionally, a hint signal composed of two units ($H = 2$) encoding for the Boolean locked variable (square signal active when true) is injected on top of the expert trajectories (via a Gaussian random projection matrix of zero mean and variance σ_{hint}) before computing the target activity.

The agent receives as input the difference of both button's and food's position with respect to current agent location, the input vector is then represented as $\Delta^t = \{\Delta x^t_b, \Delta y^t_b, \Delta x^t_f, \Delta y^t_f\}$. This vector is then encoded via a set of tuning curves: the domain is partitioned in each direction (x and y) with a resolution of 20 cells, each cell coding a scalar input via a Gaussian activation centered around it physical location and of unit variance (with a peak value of 1). Thus, each of the four input vector components $\Delta x^t_{b,f}, \Delta y^t_{b,f}$ are encoded using 20 units, yielding a total input count of $I = 80$ units. The agent output is the velocity vector $v_{x,y}$, encoded using $O = 2$ output units. The agent reward is computed as $r_{\text{unlock}} = \min\, d\left(p_{\text{agent}}, p_{\text{target}}\right)^{-1}$ when the target is successfully unlocked (button pushed), while fixed at $r_{\text{locked}} = 1/d_{\max}$, $d_{\max} = 5$ for the locked condition. The feedback matrix D_{ik} is computed as $\mathsf{D} = \mathsf{R}^\top \mathsf{R}$, where R is a Gaussian random matrix of shape $\mathbb{R}^{\mathcal{D}\times N}$, with $\mathcal{D}$ the predefined matrix rank, and variance $\sigma = 1/\sqrt{\mathcal{D}}$. The learning procedure is the same as described in Appendix B.1. We trained our system for 1000 epochs (with Adam optimizer) and repeat the experiment 100 times for each rank.

C.2 Random R matrix

We report here results obtained for the pure non-clamped version of the rule (24) and random feedback matrix R_{ik}. In Fig.SC we report the results of the analysis. In Fig.SCA the average reward (error bars omitted for visualization clarity) as a function of the test angle θ is plotted for the different ranks of the feedback matrix D, highlighting the superior performances of the high-ranks for this particular task. This features is then confirmed in panel Fig.SCB, where the average reward (across all test conditions) is reported as a function of the rank $\mathcal{D}$ of the feedback matrix D, again stressing how a high-rank feedback induces optimal performances for this task. This result confirms that what reported in the main text for a diagonal feedback matrix R_{ik} is indeed general and holds for a random R_{ik} as well. The complete set of parameters used in the Button & Food task is reported in Table B.

	Network Parameters	
Name	Description	Value
N	Number of units	300
T	Expert demonstration duration	80
I	Number of input units	80
O	Number of output units	2
H	Number of hint units	2
dt	Time step	1ms
τ_m	Membrane time scale	6 dt
τ_s	Synaptic integration time scale	2 dt
τ_{RO}	Readout time scale	10 dt
$\tau_\star$	Target time scale	5 dt
δv	Pseudo-derivative width	0.01
v_{rest}	Rest membrane potential	-4
	**Training Parameters**	
σ_J	Variance of initial weights	$1/\sqrt{N}$
σ_{input}	Variance of input matrix	5
σ_{teach}	Variance of teach signal	3
σ_{hint}	Variance of hint signal	5
η	Recurrent learning rate	0.01
η_{RO}	Readout learning rate	0.01
σ_{D}	Variance of feedback matrix	$1/\sqrt{\mathcal{D}}$
epochs	Training iterations	1000

Table B: Parameters for the Button & Food task

D 2D Bipedal Walker

D.1 Training details

The 2D Bipedal Walker environment was, provided through the OpenAI gym (`https://gym.openai.com` [3], MIT License). The expert behavior is obtained by training a standard feed-forward network with PPO (proximal policy approximation [4], in particular we used the code provided in [1], MIT License). The average reward performed by the expert is $\langle r \rangle_{\mathrm{exp}} \simeq 180$ while a random agent achieves $\langle r \rangle_{\mathrm{rnd}} \simeq -120$. The sequence of states-actions is collected in the vectors $y_k^{\star\ t}$, $k = 1, \dots O$, $x_h^{\star\ t}$, $h = 1, \dots I$, $t = 1, \dots T$. The learning procedure is the same as described in Appendix B.1. All the parameters of the experiment are reported in Table C. For this experiment, we chose the maximum rank ($\mathcal{D} = N$). In this case, if the matrix R_{ij} is extracted randomly (e.g. Gaussian with zero mean) the matrix D_{ij} is almost diagonal. For this reason, we take $\mathsf{D}_{ij} = \delta_{ij}$. We evaluate the performances for different values of $\tau_\star$. The learning is divided in 2 phases. In the first one, the recurrent weights are trained in order to reproduce the internal target dynamics $s_i^{\star t}$ for 500 iterations using gradient ascent. In the second phase of the training procedure, only the readout weights are trained using equation eq.(20).

Table C: Parameters for the Bipedal Walker 2D

Network Parameters		
Name	Description	Value
N	Number of units	500
T	Expert demonstration duration	400
I	Number of input units	15
O	Number of output units	4
dt	Time step	1ms
τ_m	Membrane time scale	4 dt
τ_s	Synaptic integration time scale	2 dt
τ_{RO}	Readout time scale	2 dt
δv	Pseudo-derivative width	0.2
v_{rest}	Rest membrane potential	-4

Training Parameters		
σ_J	Variance of initial weights	0
σ_{in}	Variance of input matrix	2.0
σ_{teach}	Variance of teach signal	3.0
η	Recurrent learning rate	0.3
η_{RO}	Readout learning rate	0.00375
epochs	Training iterations	500

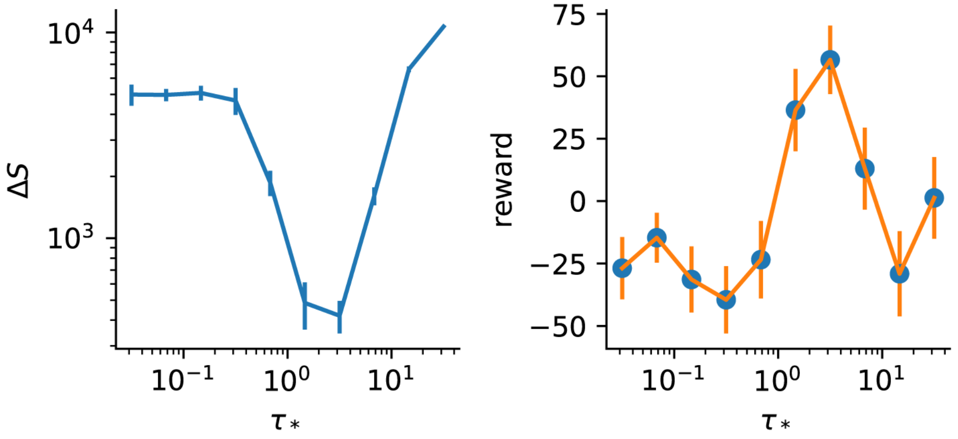

Figure D: **Bipedal Walker 2D. Non clamped version.** (left). Spike error ΔS as a function of the $\tau_\star$. (right). Average reward as a function of the $\tau_\star$.

D.2 Non-clamped case

In the experiment reported in Fig.5 of the main text we adopted the clamped version of the learning rule. This means to substitute the target spiking activity $s_i^{\star t}$ to the activity produced by the network s_i^t in the evaluation of the spike response function e_j^t.

We repeated the experiment in the non-clamped version of the training. In Fig.SD it is reported the average of the maximum reward as a function of $\tau_\star$. For each value of $\tau_\star$ we performed 10 independent realizations of the experiment. For each realization, the $s_i^{\star\ t}$ is computed, and the recurrent weights are trained. The optimization of the recurrent weights is performed using eq.(24) through gradient ascent and a learning rate η. The learning of readout weights is performed using eq.(20) through gradient ascent and a learning rate η_{RO}. Every 75 training iterations of the readout training, we test the network and evaluate the average reward $\langle r \rangle$ over 50 repetitions of the task. We then evaluate the average over the 10 realizations of the maximum $\langle r \rangle$ obtained for each realization.

It is apparent that in this case there exist an optimal $\tau_\star \simeq 2.5$ms, allowing for a minimal training error ($\Delta S = \sum_{it} |s_i^{\star t} - s_i^t|$, the difference between the target pattern of spikes and the pattern generated is minimal, Fig.SD left panel) and a maximum value of the reward (Fig.SD right panel). However, the relationship between the training error and the reward is not trivial since for high $\tau_\star$ value we got a very poor training error, but also a good value of the average reward.

Following the feedback matrix construction described in section 2.3.1 of the Numerical Results, we generalized the non-clamped experiments for arbitrary rank R, allowing for an exploration of the $(\mathsf{R}, \tau_\star)$ plane (similarly to Fig.2 of the main text), with the additional merit of testing the resulting trained model in a closed-loop environment. We selected two rank values ($\mathsf{R} = 4$ and $\mathsf{R} = 500$, corresponding to the error-based and target-based limit), and three values for $\tau_\star/dt \in \{0.1, 3.0, 10\}$. The results of this experiment are reported in Fig.E. In Fig.EA we measured the trained model performance (total final episode reward) in the Bipedal Walker 2D environment as training progresses: the model is trained to reproduce a target expert behaviour and every 150 training epochs we test it in a closed-loop fashion in the environment and record the total episode reward. Thin lines in Fig.EA are individual experiment, while the thick line and corresponding shading mark the average trend with its corresponding standard deviation. This results agree with Fig.D, clearly highlighting an optimal $\tau_\star$, while little to no dependence from rank R is detected. In the panels of Fig.EB we report the output-error (measured as MSE) and spike-error ΔS for the six explored configurations. Here again we recovered what has been reported in Fig.2 of the main text: high-rank enables low spike-error, with an optimal $\tau_\star$ for corresponding low output-error, while the low-rank version fails convergence to spike-pattern and enjoys higher $\tau_\star$ for better output-errors.

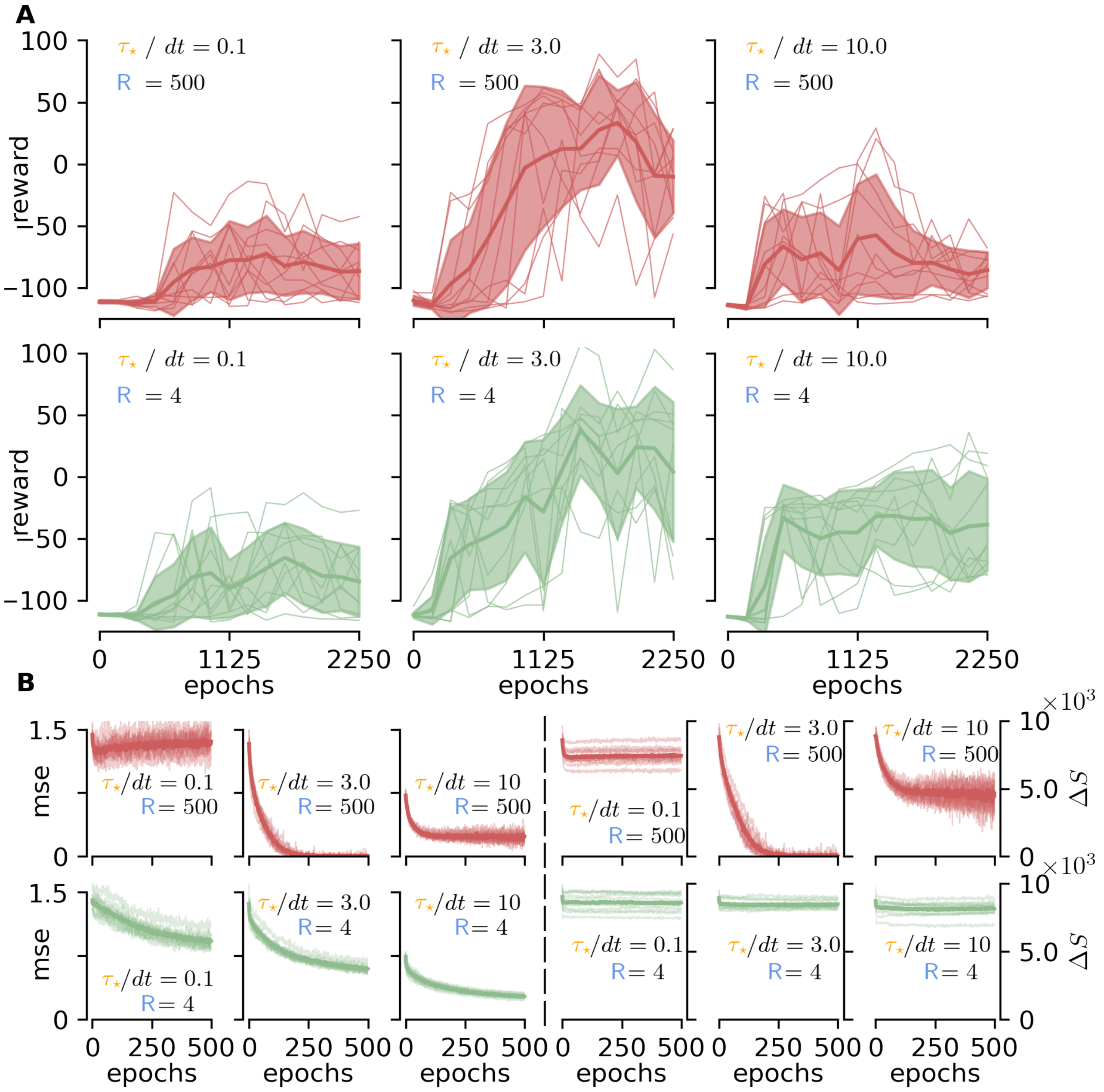

Figure E: **Bipedal Walker 2D. Closed Loop Results.** (**A**) Results for the (closed-loop) measured final episode reward for different rank R and $\tau_\star$ configuration, as a function of the training epochs. The model clearly displays an optimal $\tau_\star$ configuration for intermediate values, with the rank structure R having negligible effect on the closed-loop performance. (**B**) Training metric (output-error measured as the MSE and spike-error ΔS) for the six parameter configurations tested in panel **A**. We find similar results as those reported in Fig.2 with high-ranks achieve low spike-errors for intermediate $\tau_\star$.
