# Supplementary material for: Error-based or target-based? A unified framework for learning in recurrent spiking networks
Source: PLoS Comput Biol. 2022 Jun 21;18(6):e1010221. doi: 10.1371/journal.pcbi.1010221 (PMC9249234; doi:10.1371/journal.pcbi.1010221)
Supplement: Attachment — Submitted filename: PCB - Reviewers Reply.pdf [file pcbi.1010221.s003.pdf]

Reply to the Reviewers

Error-based or target-based? A unifying framework for learning in recurrent spiking networks

C. Capone*, P. Muratore*, P. S. Paolucci

Reviewer #1: The manuscript has been improved considerably.

However, we feel that it is still necessary to improve the clarity of exposition.

Practically, the authors propose an interesting analysis and methodology to expand the target dimensionality to control the number of constraints that the network is subjected to. The expansion of the target is random and accomplished by adding random values in the definition of the 'read-out' matrix. The fact that this expansion can help

the learning process to discover a better solution is interesting per se. We feel that this result should be clearly introduced as one of the main contributions of the work.

The idea is clearly fascinating and the analysis is novel, but it is still not simple to understand the complete training process.

We advise the authors to describe, point by point, how learning is accomplished.

After careful reading:

- First, the target signal has been processed by the untrained network.

- Then, the read-out has been trained to reproduce the target signal.

- The output connectivity matrix is expanded to include possible constraints.

- Finally, such an output connectivity matrix is adopted in the learning process to adapt the recurrency.

We think it would be better to describe this methodology clearly in one section and to justify the different choices.

We included a new dedicated section where this procedure is explained in detail.

Since the core of the work lies in the possibility to add constraints, thanks to which it is possible to move in the target/error-based spectrum, we suggest that the authors should introduce the different tasks by saying explicitly what the number of constraints vs the dimensionality of the target is. This can be inferred and it was explicit in your previous response, but saying it also in the text would help the reader.

We defined the dimensionality of the target O and of the feedback R in every task.

We have also some comments on the figures:

- Fig. 1D-E. How is this figure generated? The authors should clarify if this is an intuitive explanation of the different regimes of learning or if it was generated quantitatively.

The regime where tau/delta_t<<1 would correspond to a simulation where the discretization step is greater than the characteristic time, and I am unsure how this could be practically achieved with numerical methods. Can you clarify this point?

According to equation(1) when the timescale is very small \bar s = s (the temporal filter leaves the signal unchanged).

We added one sentence in the text to clarify this:
“Indeed, when $\tau_{\star}$ is small $\bar s_i^t \simeq s_i^t $, and it can assume only values 0 or 1.”

Also, the ideal space represented has \delta s_1 and \delta s_2 as x and y-axis, but the line corresponding to the error-based regime is increasing until it reaches the target-based limit for a maximum rank. In that limit, \delta s\approx 0. I would have imagined the target-based limit on the left approximately at zero, and the error-based limit on the right. Increasing the rank would correspond to going from right to left in this case, etc. Probably, the intended ideal x-axis is the rank.

The two axes (x and y) represent the errors (difference between network activity and the target) for 2 sample neurons (DS1 and DS2) at a specific time t.

If one considers an error-based learning rule, the optimal solution (when the MSE=0) is defined by the equation \sum_i B+_i (Delta s_i*^t) = 0, which can be represented as a line (the green line in Fig.1D-E) in the (DS1, DS2) plane.

On the other hand, the target-based solution univocally defines one solution DS=0, that is a point (the red point in Fig.1D-E) in the (DS1, DS2) plane.

The low rank is equivalent to looking for a solution close to the green line, while the high rank is equivalent to looking solution close to the red point.

Also, what are the circles? Do they simply reflect the density of the possible solutions? I would avoid writing 'good' or 'bad'.

The green circles are error-based solutions close to the green lines, accessible to the network dynamics (indeed, the spiking network activity is discretized). If the point is close to the line, it allows for a better (which we call good) solution, since the MSE is lower.

I feel that some clarifications are needed, but these panels are surely an interesting addition to the paper.

We changed the text to make this point clearer.

- Fig.2 C I can understand that the target-based limit recovers the correct succession of spikes more accurately than the error-based regime, but I am not sure that in this way the

result is meaningful. Simply, the error-based regime is characterized by a low-rank matrix and can not find the correct multi-dimensional succession of spikes. If I understood correctly, the result is an immediate consequence of the methodology adopted. Perhaps, it would be interesting to see if the error-based regime can recover the succession of spikes from the 'original' target, neglecting the additional constraints.

We believe that this question is already answered in the panels Fig.2B-C.

Fig.2B shows that both high and low-rank rule, allows for finding a good, solution, providing an output y, that is close to the target output y* (the panel shows the MSE between y and y*).

At the same time, Fig.2C shows that for high-rank the internal dynamics converge exactly to the internal proposed target s*. On the other hand, when the rank is low, this does not happen, and the network autonomously finds an alternative internal dynamics, that is different from s*, but still produces an output y similar to y*.

We acknowledge that this was not clear in the text, and we added a sentence to clarify it.

Minor:

The text has typos, please do extensive proofreading.

Fig.2A, please consider using dots for the target to make it visible.

We fixed it.

Fig.2 D-E-F, please fix the labels on the x-axis.

We fixed it.

Reviewer #2: the authors have replied to my comments and I therefore recommend the paper for publication.

Reviewer #3: The authors have done a great job in addressing my concerns in the revision of the manuscript.
