# Supplementary material for: Error-based or target-based? A unified framework for learning in recurrent spiking networks
Source: PLoS Comput Biol. 2022 Jun 21;18(6):e1010221. doi: 10.1371/journal.pcbi.1010221 (PMC9249234; doi:10.1371/journal.pcbi.1010221)
Supplement: Attachment — Submitted filename: PCB - Reviewers Reply.docx [file pcbi.1010221.s002.docx]

**Reply to the Reviewers**

**Error-based or target-based? A unifying framework for learning in recurrent spiking networks**

**C. Capone*, P. Muratore*, P. S. Paolucci**

Reviewer's Responses to Questions

Comments to the Authors:

Please note here if the review is uploaded as an attachment.

**Reviewer #1**: The authors propose an interesting model to unify target and error-based learning rules.

The proposed framework defines a spectrum of learning regimes thanks to the manipulation of the number of constraints imposed during training.

Such a number of constraints is directly defined thanks to the rank of the error propagation matrix.

The idea underlying the work has the potential to constitute a relevant contribution to the field.

However, the manuscript should be restructured to be clearer and more simulations are needed.

Major:

1 - The first main concern regards the tasks adopted to demonstrate the advantages of the framework.

For the first task, a high rank of the error propagation matrix seems to be beneficial. For the second application, the importance of the rank is not shown and the authors state that it is almost irrelevant.

Thus, the work demonstrates the advantage of a target-based learning rule, and a successful example of the framework when the system is closer to an error-based regime is missing.

The lack of this demonstration negatively impacts the applicability of the framework and the understanding of the reader.

At the end of the paper, the reader is left wondering why the model, whose aim is to provide a unification between the two learning paradigms, has been exploited only in the target-based regime.

Thus, I suggest demonstrating some advantages of the system when the rank of the matrix does not correspond to the number of nodes.

If the latter is not achievable, the authors should clearly explain why their model can still offer advantages in comparison to other published alternatives.

For instance, this could be accomplished by comparing the performance of the model proposed with e-prop, LTTS, or full-FORCE.

The convergence time of the model could be also reported for the different cases explored, providing an additional metric to evaluate the performance in the different regimes.

1- We thank the reviewer for the precious comments. The major value of our framework is the possibility to reconcile different learning approaches, allowing for explaining the difference and the similarities between them.

To better explore this point, we exploit the store and recall task described in the main text.

We do this by using the original generalized formulation (eq.(8) of the new manuscript, see the section 2.3.1 for details on the implementation) without taking the target-based limit.

We task the network to reproduce a target 3D trajectory, given a clock-like input (panel A- bottom of the figure below, dashed line: target output, solid line: network output at the end of the training for low and high-rank conditions, green and red respectively). In panel B we show that the MSE decreases during training, showing the effectiveness of the training procedure. The final MSE is lower for high rank. Interestingly, the convergence is faster for low rank case.

We now define the spike error as ΔS = sum_{it} |s_i^*t - s_i^t |. We demonstrate that the high-rank case is equivalent to the target-based formulation, since ΔS goes to zero at the end of the training, and the internal dynamics converges to the target one (panel C). On the other hand, the low-rank case does not show convergence to the target internal dynamics s*.

Finally, we provide an extensive exploration of the performances in the plane (R, tau*). MSE, ΔS, and the convergence timescale (defined as the time required to halve the initial MSE) are color-coded and respectively reported in panels D-E-F. This exploration evidences the presence of an optimal tau* that minimizes the MSE and the ΔS.

We report these new results in Fig.2 of the new version of the manuscript.

However, these results do not directly apply to closed-loop tasks, in which learning with a very low error is not always optimal, and it could bring to overfitting conditions (e.g., the network learns a very specific condition, and poorly performs when tested on validation conditions.)

For this reason, we also applied the same approach described above in a closed-loop task, using a behavioral cloning framework. We use a supervised learning approach to replicate the demonstration of an expert, given a specific input. Our framework allows exploring the result for different values of rank R and tau*.

As a first step, the recurrent weights are trained using the learning rule in eq(8). We repeated the training for different values of rank (4 and 500) and tau (0.1, 3, 10)ms. Similar to the previous experiment, for high rank, the MSE decreases while the internal dynamics converges to a specific s* (see the updated section D.2 of the Supporting Information for further details).

In a second phase, read-out weights are trained. In the figure below, it is reported the average reward as a function of training steps (for different R and tau* values). In agreement with our previous results (see section D.2 of the Supporting Information), the performances of this task do not depend on the rank but are very sensitive to the tau*.

We report these new results in the supporting information (section D.2).

2 - Relative to the above, it would also be important to show what is the impact of the additional constraints Y_{k, init}. A number of targets equal to N can be set by simply using the untrained network, and it would be beneficial to know what is the value of O (Eq. 10) in the different cases.

2 - We believe that the part of the question regarding the effect of the additional constraints was already addressed in the previous answer. Indeed, we explored the performances of the model in the tasks ‘store and recall a 3D trajectory’ and the ‘2D bipedal walker’ as a function of R (the number of constraints).

In the store and recall task, O = 3 (the number of target output trajectories). For the button and food and the 2D bipedal walker O is 2 and 4 respectively.

It emerges that a number of constraints equal to N is equivalent to constraining the dynamics of each neuron of the network, since the internal dynamics converges to an internal target s*.

However, we acknowledge that the definition of the additional constraints q and of the generalized target output should be refined. For this reason, we created a new section (2.2.2) where this aspect is described in more detail.

To summarize, the first O components of Y*_k are the same as t y*_k, while the additional constraints q*_k are a random linear combination of a hypothetical internal solution s*_i. As discussed in the text, s*_i is the response of the untrained network to the target output y*.

This specific definition allows for controlling the convergence of the internal dynamics, even using the general learning rule eq.(8).

3 - The paper is difficult to read and should be structured differently. In general, the paper feels fragmented and multiple sections could be merged.

The authors should introduce the neuronal model and the network architecture used, and then introduce the learning rule adopted.

3 - We agree with these suggestions, we implemented such modifications in the main text. We also merged and renamed other sections to improve linearity and readability.

4 - The additional constraints, which are fundamental for the work, are briefly explained in 2.1.5, while other targets are introduced in Section 2.1.4 by using an untrained network with the desired output as input similarly to the full-FORCE algorithm. The complete set of constraints is unclear.

I would suggest explaining the overall model in one section and drawing an explicit diagram that depicts also the untrained network, the different targets, and where these are coming from.

After such an overall definition, the authors could describe the different learning regimes. Also, it is important to describe how the system is initialized since the untrained network is used for learning.

4 - We agree that the additional constraints are a crucial point in our work. For this reason, we introduced a new section (2.2.2) where we discussed this point in detail.

Also, we confirm that, similarly to the full-FORCE algorithm, the definition of s* depends on the initialization of the network. In our case, we initialize it with null recurrent weights. We clarified this point in the text.

In order to give a more immediate understanding of the different learning regimes, we made an effort to provide a geometrical interpretation of our model, that intuitively explains what is the role of the two parameters R and $\tau_{\star}$ in defining the network internal solution to a task.

When the rank is high, the only possible solution is the target-based solution and the learning approaches to a specific internal solution for network dynamics (panel D, red circle).

On the other hand, the error-based rule defines a set of possible solutions (defined by $\sum_i B+_h (s_i*^t - s_i^t) = 0) distributed in a subspace of possible internal states (panel E, green line) in which the MSE is low.

However, not all the points on the green line are accessible to the network (given the discrete nature of the spiking activity, see panel E black crosses), and the error-based solution achieved during the learning procedure can be sub-optimal (see panels D-E of the figure below, the green point which is far from the green line).

When tau* is large, the signals produced by the network are filtered, and the possible values are no longer either 0 or 1. As a result, the accessible states are denser in the space of possible internal solutions (see panel E, black crosses), and it is easier to find a good solution with an error-based approach (see panel E, green point, which is now closer to the green line).

This theoretical prediction is confirmed in numerical experiments (panel F), showing that the MSE is much lower for the target-based approach for low tau*, but they converge to the same value for large tau*.

Minor:

5 - Fig.1 D should be explained in more detail since it provides information on how the model fits in the literature.

5 - We introduced a new section (2.2.4) that describes in more detail this panel and the relationship with other models in the literature.

6 - The notation needs to be unified across the paper. For instance, before the constraints are called q* and then later the authors have introduced Y_{k, init}.

6 - We rewrote the text on the definition of the additional constraints (see the new 2.2.2 section) and the notation has been unified.

7 - The statement "The first..." at the end of Section 2.1.5 is unclear.

7 - In the new 2.2.2 section, we clarified this point.

8 - Section 2.2.2 and 2.2.1 could be merged, and the title of section 2.2.2 removed. I suggest explaining the second "noisy" methodology to estimate the dimensionality of the solution as an alternative, and to give minor emphasis to biological data. In the end, biological data have not been used in the paper. Instead, the authors could introduce this alternative approach and its advantages (since repetitions are not needed) and conclude with a note on biological plausibility.

8 - We agree with the suggestion and implemented it in the text.

9 - Fig. 3 is almost obvious and could go to Supplementary Material. In contrast, Fig. 1 D shows how different models adopt diverse characteristic times. The authors could comment on this and provide some understanding of the relation

between timescale and model. Are the target-based models more flexible? Is the framework proposed more flexible to changes in the characteristic time?

9 - We accepted the suggestion to move Fig3 in the Supporting Information.

We acknowledge that the understanding of the role of the different time-scales is crucial to the paper. For this reason, we included a new sketch in Figure 1 (Fig1D-E), and introduced a new figure (Fig2 of the new text).

The new Fig1D-E explains the role of the time-scale tau* in the learning rule (this point is already addressed in the reply to question 4).

In Fig.2 we extensively explore the effect of different values for the parameter tau* in the performances of the store and recall task (as described in the reply to question 1).

**Reviewer #2**:

1 - This an interesting study that makes a substantial contribution to the topic of learning in the context of network dynamics. The authors study the differences between error-based and target-based approaches, both analytically and numerically, also applying their approach to two ‘real-world’ tasks. I believe that this work can be of interest to a relatively broad readership, such as the network science community, or researchers studying learning, to name a few. Nevertheless, I still have a few small comments for small improvements that I go over below. Also, although the text is understandable, I would advise to have the manuscript proofread by a native English speaker before publication. Likewise, abbreviations used should be initially defined (e.g. the use of ‘LTTS’).

1 - We thank the reviewer for the comment and did our best to improve our English.

2 - Introduction:

As this paper is aimed at a relatively broad and not specific audience, I think it would be helpful for the reader if the topic was introduced a bit better. For instance, I don’t understand what this sentence comes to say “When first confronted with reality, humans learn with high sample efficiency, benefiting from the fabric of society and its abundance of experts in all relevant domains.”

Are you referring to babies in this case? If so, do they benefit from experts in different domains? Or do you simply refer to its’ parents?

2 - With this sentence, we wanted to stress the importance and the generality of imitation learning. In society, this is true every time an individual learns from other, more “expert” individuals.

We chose a minimal solution and removed the word “first” from the sentence.

3 - “Moreover, we observe that spike-timing-based neural codes are experimentally suggested to be important in several brain systems”

You mention ‘we observe’ - perhaps this is a minor detail, but you go on to cite other papers, so this isn’t your observation. Also, I think it would be helpful to explain what ‘spike-timing-based neural codes’ are and why they are important. The connection between this part and the rest of the intro isn’t clear and it comes a bit out of nowhere. I think it should be a paragraph on its’ own, with an explanation of the motivation for this section of the paper.

3 - We thank you for the suggestion and replaced “observe” with “remark”.

Spiking networks have the huge advantage of being able to implement precise temporal coding. However, many models don’t exploit such a feature and don’t use spikes to implement temporal coding. The advantages in models of coding with specific spike timing have been shown in papers as [29,30].

However, it is not obvious that precise spike time coding is implemented in every region of the brain. This parameter of our model serves to explore different coding regimes that might be used in different brain areas.

We made an effort to provide a further explanation of the role of \tau* in our model, which is included in figure 1. This figure was discussed extensively in the reply to question 4 of reviewer 1.

4 - Lastly, before we move on to the results section, it might be valuable to prepare the reader for what comes up next. You will explore these questions theoretically and then numerically (each with several separate steps). A clarification of the logical steps throughout could be useful.

4 - We implemented this suggestion, including the following text at the beginning of the results section:

“In the following sections, we define a generalized learning framework by identifying two sensitive parameters: the number of constraints $R$ and the sensitivity to precise temporal coding $\tau_{\star}$.

We analytically show how different learning rules presented in the literature can be accounted for as specific cases of our framework.

We provide a geometrical interpretation, and strengthen our statement through numerical experiments.

Finally, we test the performances of our learning rule as a function of the two main parameters ($R$, $\tau_{\star}$) on different tasks. A store and recall task (of a target 3D trajectory), a navigation task (button and food) and a motor task (2D bipedal walker). “

Results:

5 - As mentioned, there are a few small language issues. For instance in section 2.1.3

“propose an alternative formulations allowing to evaluate target”

Should be corrected.

5 - We corrected it.

6 - I think that the paper could either be re-structured, or, at least, that the authors would make better connections between the different sections. For instance, in section 2.2.3 you start by saying:

“As we discussed above the τ⋆”

Where is this discussed above? It isn’t anywhere in section 2.2.2. Why is this here now?

6 - We changed the structure of the theoretical part of the results; we merged sections and introduced new ones to improve linearity and readability.

Section 2.2.2 (old version) was moved to the Supporting Information as suggested by reviewer 1.

7 - In the experiment section, many choices are made by the authors. Although this is of course necessary, no explanation for the reasoning behind these choices are given (e.g.:

“The readout time-scale is fixed to τRO = 5ms while the readout weights are initialized to zero and the learning rate is set to ηRO = 0.01.”

Why are these values chosen? I think there should be some kind of ‘sensitivity analysis’, showing that similar results would be gained with slight variations of these parameters, or, at least, giving justification for them.

7 - The choice of hyper-parameters is usually a delicate point.

For example, the learning rate (ηRO) should be picked not too small to speed up learning, but not too large, or the gradient-based optimization would not work.

Here we report the results on the store and recall for three different η (the learning rate of the recurrent weights). In the three cases, the MSE is reduced over training. However, the smaller η value (black) is too small, and the convergence is slow. The larger η value (orange) allows for faster convergence, but the convergence to the optimal value requires time. The intermediate η value allows for optimal convergence of the learning procedure.

In this task, the parameter τRO is extensively investigated. Indeed, in the store and recall task, τRO is the same as tau*. The panel below shows the MSE between the output and the target at the end of the training procedure. In this case, it exists an optimal timescale that minimizes the error (5ms for high ranks, and around 20ms for low ones). This panel is part of the new figure 2.

Discussion:

8 - The discussion is a bit disappointing, as it is very brief and doesn’t really engage with the literature. If this work isn’t placed adequately within the existing studies, it can loose much of its relevance. Also, there is no discussion with regards to the limitations of this study.

8 - We introduced a new section “limitations of the study”, commented on the generality of the framework (at the beginning of the discussion) and included new references.

Moreover, the new section of the results 2.2.4 describes the relationship between our framework and the existing learning approaches.

9 - On a more specific note (and perhaps this is a bit outside the usual scope of the field), I think that it would be interesting to consider, and discuss, the consequence of non-experts in the vicinity of the agent. In this paper, you assume that the agent learns from experts that surround it. This is a fair starting assumption, but in reality we usually have heterogenous populations where actually there is a chance of learning undesirable traits (by imitation).

9 - In the behavioral cloning framework, one assumes that the agent clones only the behavior of an expert demonstrator, whose policy is nearly optimal by definition. However, it is possible to consider demonstrations that are suboptimal taking into account different learning frameworks, such as q-learning from demonstrations, where an explicit reward is associated to the specific action of the demonstrator (see the reference below).

[Hester, Todd, et al. "Deep q-learning from demonstrations." *Proceedings of the AAAI Conference on Artificial Intelligence*. Vol. 32. No. 1. 2018.]

Figure 3:

10 - I think that in subplots A and B it would be better to name the x-axis ‘number of iterations’.

The caption of Panel C reads “Scatter plot of mse vs ΔS for different values of τ⋆.” But on the plot itself we have the absolute value of ΔS. This should be resolved. Also, this isn’t a scatter plot. This is also color-coded, but compared with plots A and B, this isn’t mentioned. As the color coding is equivalent in all three, this can be mentioned once for the whole figure.

10 - We implemented the corrections. The figure was moved in the supplementary material, as suggested by reviewer 1.

Figure 4:

11 - Subplot C isn’t helpful in its’ current form. Either show less examples, colour-code them differently (keeping everything blue makes it harder to distinguish), or expand the size of the plot. We fixed it.

For subplot D please write explicitly what the dashed and solid lines are. We fixed it.

In Panel G - what do the error bars show? Please state this explicitly. Also, what is the color gradient? Same as in F? If so it should be placed on the right of G, or G should have its own. We fixed it.

11 - We rearranged the figure to make it clearer and more readable.

Figure 5:

12 - Subplot B should be split into two (as it has two subplots). So the bottom one should have its own letter referring to it.

Subplot D has the same problem as Fig4C.

12 - We rearranged the figure to make it clearer and more readable.

Reviwers conclusion:

In conclusion, I think this is a nice and insightful piece of work, and I would therefore recommend it for publication after minor revision. As the changes suggested are minor, the editor may decide to accept them without my further involvement. But I am also happy to check the revised version of this manuscript.

**Reviewer #3**: The authors introduced a general framework for supervised learning in recurrent spiking networks, with two main parameters, the rank of the feedback error propagation and the tolerance to precise spike timing. And they analyzed the learning performance for different parameters. The research topic is very important for not only constructing efficient machine learning methods but also understanding biological neural networks. However, I have some concerns below.

Major comments

1 - While error-based learning is familiar, target-based learning is a bit unfamiliar to many readers. Therefore, I recommend that the authors add more explanations about target-based learning in the Introduction. Also, they should describe the critical differences in the characteristics and learning performances between the two. Moreover, LTTS and full-Force, which appear in Results, should be explained briefly.

1 - We thank the reviewer for the comment, we acknowledge that this is an essential point and that it is not clearly explained in the text.

For this reason, we introduced a new section, “2.2.4 Interpretation of the model” where we address this point.

2 - The authors state that ‘Moreover, our general framework, encompassing different learning formulations, allowed us to investigate what learning method is optimal to solve a specific task.’ in the Discussion. However, only two tasks, the button-and-food task and the 2D Bipedal Walker, were used for checking the performance in the manuscript. It seems that the best performance was achieved at a higher rank of error propagation matrices. What kind of task favors a lower rank of error propagation matrices? After all, the higher the rank of D, the better it is? If so, the claim ‘different brain areas might be located in different positions in the plane presented in Fig.1C’ is invalid for the rank of D (error-based or target-based learning).

2 - We thank the reviewer for raising this very interesting point. To answer this question, we extensively explored the “store and recall of a 3D trajectory”. Here we investigate the performance of our approach, in the plane (R, tau*). We observe that, while high rank allows for lower MSE, a low rank allows for a faster convergence, in agreement with what observed in previous works (LTTS [30]). We can conclude that a network committed to perform a memorization task could benefit from a low rank error propagation matrix.

These new results have been described in detail in the reply to the question 1 of the reviewer and now reported in figure 2 of the new version of the text.

Minor comments

3 - Clarify the definition of \delta_t and \tau_star in Eq. (2).

3 - We clarified the definition of the two parameters.

4 - Why is the star mark in \tau of Eq.(2)? It’s confusing to me because it looks like a desired time scale parameter.

4 - Our idea is to use the super/subscript * for the variables concerning the target quantities. tau* is the temporal tolerance to the encoding of the target internal solution s*. We clarified this in the manuscript by introducing the following text:

“We interpret the parameter $\tau_{\star}$ (the time-scale of the spike filtering as the tolerance to spike timing of the proposed internal solution $\bar s_i^{\star t}$. This clarifies the use of the subscript $\star$ for the timescale $\tau_{\star}$, since it concerns the target quantities.“

5 - Fig. 4F: We cannot see the error bars in F, but there is a description of error bars in the caption.

5 - Fixed.

6 - Fig. 4G: We can see the messy graph around 300.

6 - Fixed.

7 - In p.9, Fig.5D, Fig.5G -> Fig.4D, Fig.4G

7 - Fixed.

8 - What’s the reward? The performance r?

8 - In closed loop tasks, the performance is usually measured as the total reward obtained at the end of the episode.

We see that this point is not clear in the button and food task. We included a new piece of text specifying that the performance is measured through a reward function r and better defining the reward function itself.
